# Nuclear translocation of the tagged endogenous MAPK MPK-1 denotes a subset of activation events in *C*. *elegans* development

**DOI:** 10.1242/jcs.258456

**Published:** 2021-09-03

**Authors:** Neal R. Rasmussen, David J. Reiner

**Affiliations:** Institute of Biosciences and Technology, College of Medicine, Texas A&M Health Science Center, Texas A&M University, Houston, 77030, USA

**Keywords:** EGF, EGFR, LET-23, LET-60, LIN-45, MEK-2, MAPK, *C. elegans*, Nuclear translocation, nKTR, KTR, Vulval development, Cell fate patterning

## Abstract

The extracellular signal-regulated kinases (ERKs) are mitogen-activated protein kinases (MAPKs) that are utilized downstream of Ras to Raf to MEK signaling to control activation of a wide array of targets. Activation of ERKs is elevated in Ras-driven tumors and RASopathies, and thus is a target for pharmacological inhibition. Regulatory mechanisms of ERK activation have been studied extensively *in vitro* and in cultured cells, but little in living animals. In this study, we tagged the *Caenorhabditis elegans* ERK-encoding gene, *mpk-1*. MPK-1 is ubiquitously expressed with elevated expression in certain contexts. We detected cytosol-to-nuclear translocation of MPK-1 in maturing oocytes and hence validated nuclear translocation as a reporter of some activation events. During patterning of vulval precursor cells (VPCs), MPK-1 is necessary and sufficient for the central cell, P6.p, to assume the primary fate. Yet MPK-1 translocates to the nuclei of all six VPCs in a temporal and concentration gradient centered on P6.p. This observation contrasts with previous results using the ERK nuclear kinase translocation reporter of substrate activation, raising questions about mechanisms and indicators of MPK-1 activation. This system and reagent promise to provide critical insights into the regulation of MPK-1 activation within a complex intercellular signaling network.

## Abstract (precis)

**Summary:** Tagged endogenous *C. elegans* MPK-1 shows activation-dependent cytosol-to-nuclear translocation. This tool provides novel insights into MPK-1 localization compared with other markers of in vivo ERK activation.

## INTRODUCTION

The mitogen-activated protein kinase (MAPK) superfamily regulates a diverse series of cellular functions, including cell proliferation, migration, and differentiation (Kolch, 2005). The most well-known families are the conventional p38 MAPKs, c-Jun N-terminal kinases (JNKs), and extracellular signal-regulated kinases (ERKs; hereafter denoted ERK when used generically), all of which exhibit high degrees of conservation across metazoans (Cargnello and Roux, 2011). Early work in *Drosophila* and *C. elegans* identified orthologs of ERK – Rolled and MPK-1, respectively – as the terminal kinases of the Ras–Raf–MEK–ERK signaling cascade (Biggs et al., 1994; Wu and Han, 1994; Lackner et al., 1994). The role of MPK-1 in *C. elegans* development was first uncovered in the vulval precursor cells (VPCs) as part of the Ras LET-60 signaling cascade promoting primary fate. The ERK MPK-1 was shown to be both necessary and sufficient for proper induction of primary fate within the VPCs (Lackner and Kim, 1998). MPK-1 also has key roles in other *C. elegans* tissues, including induction of excretory duct cell fate, multiple developmental events during germline proliferation, roles in nervous system function, and immune response to pathogenic bacteria (Arur et al., 2009; Church et al., 1995; Nicholas and Hodgkin, 2004; Lackner and Kim, 1998).

The ERK MAPK cascade has continued to be one of the most well-studied signaling cascades because of its role as a promising pharmacological target for anti-tumor therapies in cancers with activating mutations in Ras, Raf or upstream receptor tyrosine kinases (Ryan et al., 2015). An understanding of ERKs and their mechanisms of activation has become essential, as targeted therapeutics for activated Ras and Raf have had limited efficacy currently and can promote increased activity in wild-type Raf (Durrant and Morrison, 2018; Hatzivassiliou et al., 2010; Poulikakos et al., 2010).

The ERK signaling module consists of a three-tier kinase cascade with multiple phosphorylation events and negative feedback loops. Kinase activation of substrates in the linear activation cascade of Raf, MEKs and ERK is largely selective, and thus can generally be considered a ‘signaling module’. In contrast, the kinases upstream of the p38 and JNK MAPKs are variable and they exhibit significant promiscuity (Krishna and Narang, 2008; Chen et al., 2011). The linearity of the Raf–MEK–ERK cascade contrasts with ERKs having a large pool of substrates (∼659) and subsequent signaling outputs (Ünal et al., 2017).

Upstream MAP kinase kinases (denoted MAP2Ks) have the unusual property of being dual-specificity kinases: they phosphorylate paired threonine and tyrosine residues adjacent to consensus docking sequences on their substrate MAPK (Derijard et al., 1995; Lin et al., 1995). To counterbalance activating phosphorylation of ERK, a series of dual specificity phosphatases (DUSPs) inhibit ERK activity by dephosphorylating both the phosphorylated threonine and tyrosine residues (Huang and Tan, 2012).

With ERK serving as the primary downstream signaling branch point, the field has largely relied on detection of its dual phosphorylation status to assay its activation via immunoblotting or immunostaining. This approach has revealed substantial complexity in the spatial and temporal expression and activation of ERK. Nuclear translocation of ERK has also been used as an indicator of its activity (Lenormand et al., 1993; Gonzalez et al., 1993), although not in model organisms. However, as active ERK and its substrates can be found both in the cytoplasm and nucleus, nuclear translocation must be interpreted with caution (Ajenjo et al., 2004; Yoon and Seger, 2006; Ünal et al., 2017). To allow for temporal analyses of ERK, real-time fluorescent reporters of activity like Förster resonance energy transfer (FRET) and kinase translocation reporters (KTRs) have been developed for both *ex vivo* and *in vivo* contexts (Harvey et al., 2008; Regot et al., 2014; de la Cova et al., 2017). These improved tools have expanded our understanding of dynamic regulation of ERK signaling, but we still are still missing a facet of the activation of ERK: detection of endogenous ERK subcellular localization in real time.

In this study, we used CRISPR/Cas9-dependent genome editing to insert sequences encoding a fluorescent epitope tag into the endogenous *C. elegans mpk-1* gene, resulting in expression of MPK-1::mKate2^3xFlag protein. Visualization of endogenous MPK-1 – at the level of the whole animal and throughout development – provides a novel tool to understand further its roles in signaling and development. This approach validates certain previous observations of MPK-1 expression, localization and activation in *C. elegans*. We observed tagged endogenous MPK-1 to be expressed broadly in every part of the animal and throughout development. We also detected consistently elevated expression in tissues in which MPK-1 activity has been described by other means. In agreement with previous findings, MPK-1 was expressed throughout the germline, showing brief nuclear localization in the most proximal developing oocyte. However, our approach also yielded unexpected observations. Upon induction, cytosolic-to-nuclear translocation of MPK-1 was observed in all six developing VPCs, not just the presumptive primary (P6.p) cell, as would have been predicted from an abundance of previous studies (Lackner et al., 1994; Lackner and Kim, 1998; de la Cova et al., 2017). Translocation in VPCs also demonstrated temporal and concentration gradients, with earliest and strongest translocation in P6.p, the VPC destined to assume a primary fate. These unanticipated dynamics suggest that mechanisms of MPK-1 activation are more complex than previously understood. Taken together, our findings demonstrate that our tagged endogenous MPK-1 is a tool that reveals novel insights into MPK-1 regulation and roles in cell signaling, complementary to those measuring phosphorylation or substrate activation.

## RESULTS

### Tagging endogenous MPK-1 via CRISPR

**Fig. 1.**
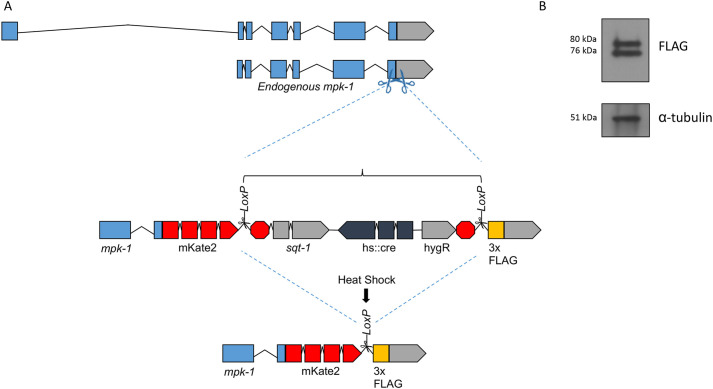
**Tagging endogenous *mpk-1* using the SEC strategy.** (A) Diagram of the strategy for the CRISPR/Cas-9-dependent knock-in of mKate2^SEC^3xFlag into the 3′ end of *mpk-1*. (B) Immunoblotting for MPK-1::mKate2^3xFlag via the 3xFlag epitope tag along with α-tubulin loading control.

The gene *mpk-1* encodes two variants with differing promoters and hence differing 5′ ends. The longer *mpk-1b* transcript is predicted to yield a protein of approximately 51 kDa, while the shorter *mpk-1a* transcript, which lacks the additional 5′ exon, is predicted to encode a protein of 43 kDa (Fig. 1A). To ensure that we tagged both isoforms of endogenous MPK-1, we elected to insert our CRISPR tag in the 3′ end of the gene, to generate proteins tagged at the C-terminus (Fig. 1A). The final tagged *mpk-1* allele was confirmed through sequencing of flanking DNA and immunoblotting against the 3xFlag epitope. Both predicted isoforms were visible as expected at 77 and 69 kDa including tags, respectively (Fig. 1B).

To confirm that our tagging strategy did not impair MPK-1 function, we examined its effects on two key MPK-1-dependent developmental events: excretory duct cell and vulval development. We observed no difference in rod-like lethality in the wild-type vs MPK-1::mKate2^3xFlag (also referred to as MPK-1::mKate2) animals at the larval (L)1 and L2 stages (Table S1). Likewise, we did not observe perturbation of vulval development in MPK-1::mKate2 animals (Table S2). These observations suggest that MPK-1::mKate2 is a functional protein that is suitable for assessing signaling activity in live animals. This reagent positions us to assay endogenous MPK-1::mKate2 functions from a novel perspective.

### Endogenous MPK-1::mKate2 is expressed ubiquitously and throughout development

**Fig. 2.**
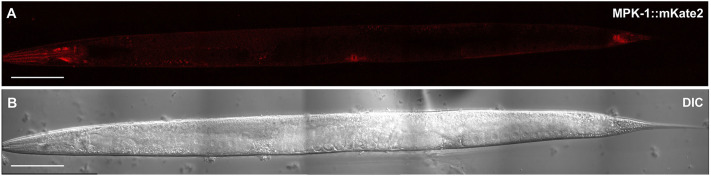
**MPK-1 is expressed ubiquitously in *C. elegans.*** Representative photomicrographs of five experiments (A) MPK-1::mKate2 expression along with (B) corresponding DIC images of an adult animal. Scale bars: 100 µm.

**Fig. 3.**
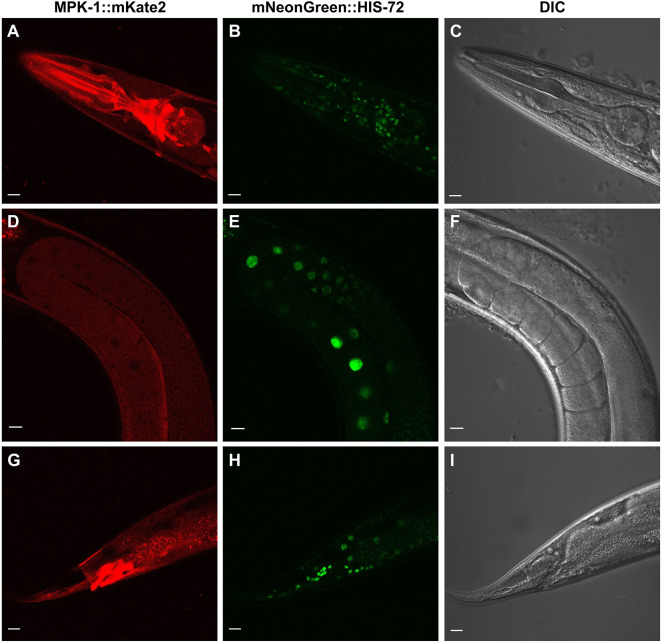
**Details of tagged endogenous MPK-1::mKate2 expression and localization.** Representative confocal photomicrographs from five experiments are shown. (A,D,G) Red filter set visualizing MPK-1::mKate2. (B,E,H) Green filter set visualizing mNeonGreen::HIS-72 nuclei. (C,F,I) DIC (Nomarski). (A,B,C) Adult pharynx. (D,E,F) Mature gonad turn without embryos. (G,H,I) Adult tail with elevated intensity in rectal epithelia. Scale bars: 10 µm.

Endogenous MPK-1::mKate2 was observed to be expressed in every cell type we could discern (Fig. 2; Fig. S1). We observed expression at each stage of development, from embryo to fertile adults, including in the mature germline (Fig. S1A–F; Fig. S2A–F). Unexpectedly, expression levels were globally elevated during the L2 and L3 stages compared to that seen in L1, L4, and adults (Fig. S1, Fig. S2). It is unclear why this change might occur and be reversed. Additionally, throughout development, higher levels of MPK-1 were observed in neurons in the head and in the rectal epithelium. We also observed elevated expression in the posterior gut, anterior gut and pharynx (Fig. S1; Fig. S2), all consistent with pro-inflammatory functions. The highest expression levels of MPK-1 were observed in the cells in the nerve ring around the pharynx and surrounding the anus (Fig. 3A–C,G–I).

Given the varied but broad expression of MPK-1 throughout the animal, we conducted a survey of its expression in various tissues in which prior research had established a role for MPK-1. Owing to the pivotal role of MPK-1 in germline development, we first examined its expression pattern throughout the gonad, where multiple MPK-1-dependent events are known to occur (Arur et al., 2009). Consistent with prior immunoblotting for total MPK-1 in dissected gonads, MPK-1 was expressed throughout the germline and generally localized to the cytoplasm (Fig. 3D–F).

### Endogenous MPK-1 is actively translocated to the nucleus in maturing oocytes

Substantial work has examined the role of MPK-1 signaling within the developing germline of *C. elegans* (Arur et al., 2009). This system has benefitted from its ability to be dissected from the animal for immunostaining to determine subcellular localization for both total MPK-1 and dual-phosphorylated MPK-1 (dpMPK-1; Lee et al., 2007). This approach has provided snapshots that allow an understanding of the spatial and temporal expression and phosphorylation patterns of MPK-1.

**Fig. 4.**
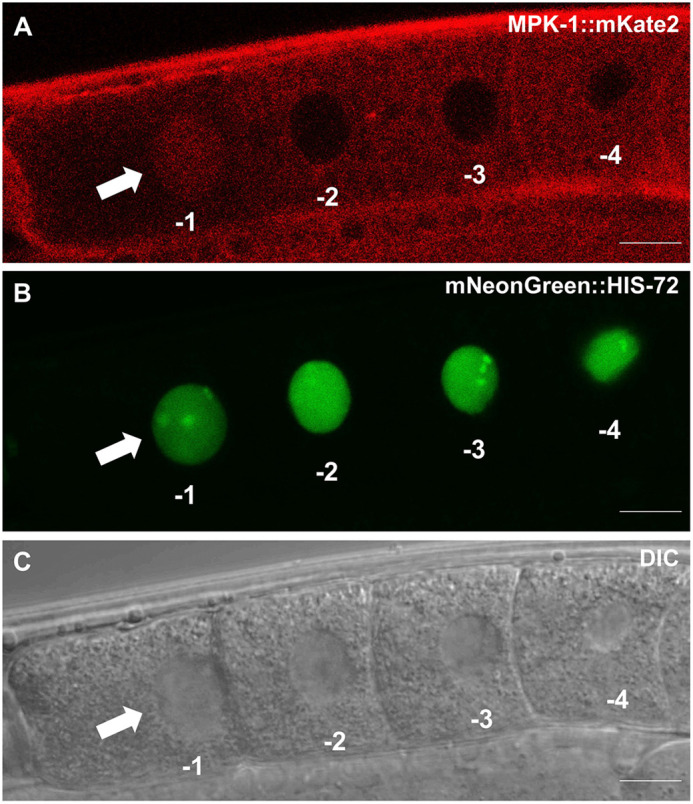
**MPK-1 nuclear localization in maturing proximal oocytes.** Representative confocal and DIC photomicrographs from 15 experiments are shown. Positions are indicated as −1 (most mature) through −4 (least mature). Arrows indicate the nucleus with translocated MPK-1::mKate2. (A) Red channel, MPK-1::mKate2 in cytosol but with a pool of tagged protein translocated to the nucleus in the most proximal oocyte (position −1). (B) Green channel, the same animals with green mNeonGreen::HIS-72 nuclear marker. (C) DIC images of the four most proximal developing oocytes. Scale bars: 10 µm.

We examined the expression pattern and subcellular localization of endogenous MPK-1::mKate2 within the proximal germline of animals 24 h post-mid-L4. In keeping with previous findings, MPK-1 expression was ubiquitous throughout the germline and largely excluded from nuclei. An examination of the four oocytes most proximal to the uterus revealed infrequent nuclear translocation of MPK-1 in the most mature oocyte (position −1). Lee et al. highlighted that dpMPK-1 staining and localization varied depending on its stage of maturation, with late-stage oocytes displaying high nuclear expression (Lee et al., 2007). We also observed this variation in the nuclear localization of tagged endogenous MPK-1 within late-stage oocytes (Fig. 4).

### RSKN-1 negative feedback regulates endogenous MPK-1 nuclear localization in developing oocytes

**Fig. 5.**
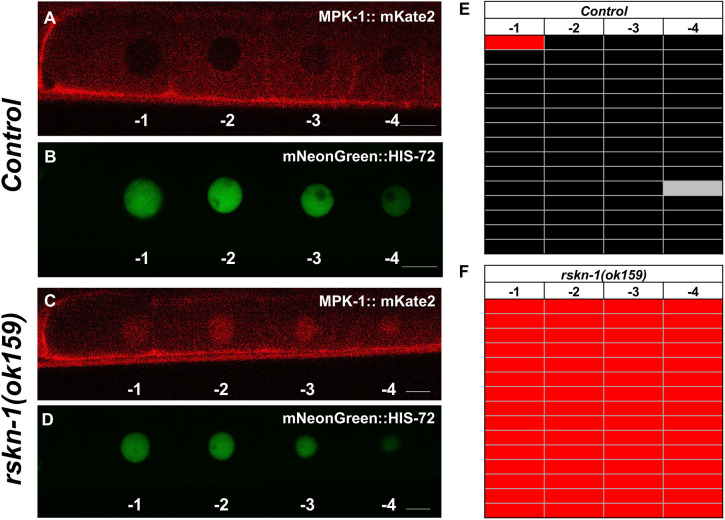
**RSKN-1 negatively regulates nuclear translocation of endogenous MPK-1 in maturing oocytes.** Matched representative confocal photomicrographs are shown. (A,B) A wild-type animal shows infrequent translocation of endogenous tagged MPK-1 to nuclei. (C,D) An *rskn-1(ok159)* deletion mutant reveals translocation of MPK-1 to every maturing nucleus. (B,D) mNeonGreen::HIS-72-marked nuclei. (A,C) MPK-1::mKate2 signal. Scale bars: 10 µm. (E,F) Tabulation of observed wild-type versus mutant animals (*n*=15). Red indicates nuclear localization of MPK-1::mKate2, and black indicates nuclear exclusion of MPK-1::mKate2; the gray marker in E represents an animal in which the nucleus in position −4 was out of the plane of focus.

Prior work has established that RSKN-1, the *C. elegans* member of the p90 RSK family (Carriere et al., 2008), is a downstream target of MPK-1 that functions in a negative-feedback loop to inhibit and/or restrict activation of MPK-1 in maturing oocytes: *rksn-1*-specific depletion by RNA interference resulted in expansion of the compartment of oocytes displaying dpMPK-1 staining (Arur et al., 2009). Building on our previous findings with nuclear MPK-1 expression in the most mature diakinetic oocyte, we compared animals with or without deletion of *rskn-1*. In the wild type, we observed consistent nuclear exclusion of MPK-1::mKate2 in the four most proximal diakinetic oocytes (Fig. 5A,B,E). In contrast, in *rskn-1(ok159)* mutant animals, nuclear localization was consistently observed in all four proximal oocytes (Fig. 5C,D,F). This result validates cytosol-to-nuclear translocation of endogenous tagged MPK-1 as a biomarker for activation.

### Pn.p cells neighboring VPCs exhibit nuclear MPK-1 prior to VPC induction

**Fig. 6.**
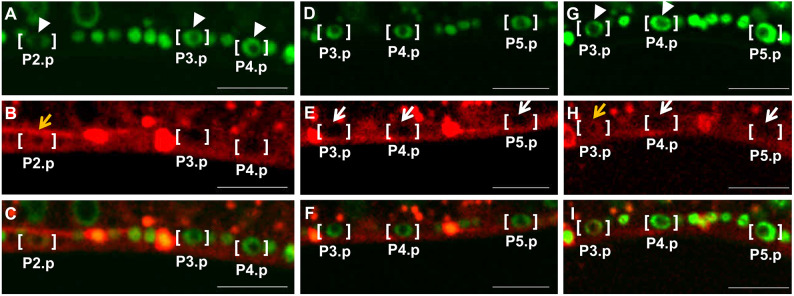
**Pn.p neighbors of VPCs have high nuclear MPK-1.** (A,D,G) mNeonGreen::HIS-72 nuclear marker; (B,E,H) MPK-1::mKate2 red signal; (C,F,I) Merged images. Square brackets indicate Pn.p cells. Large spaces of signal exclusion are nuclei, small spaces of signal exclusion are nucleoli (see green DNA signal). Orange arrows indicate high nuclear MPK-1, white arrows indicate low nuclear MPK-1. White arrowheads indicate VPC and non-VPC nuclei with differing levels of mNeonGreen::HIS-72 intensity. (A,B,C) Nuclear MPK-1 is high in P2.p and low in P3.p and P4.p. (D,E,F) Nuclear MPK-1 is low in P3.p, P4.p and P5.p. (G,H,I) Nuclear MPK-1 is high in P3.p and low in P4.p and P5.p. Images are representative of 10 experiments. Scale bars: 10 µm.

We will describe how tagged endogenous MPK-1 translocates to the nuclei of all VPCs, the classic system for analysis of MPK-1 in *C. elegans* (see below). But first we will note our observation that P2.p, which is not a VPC and thus not competent to be induced to develop as a vulval lineage, exhibits a pool of tagged endogenous MPK-1 in its nucleus at a time when its posterior neighbors and VPCs, P3.p and P4.p, do not have nuclear MPK-1 (Fig. 6A–C). We propose that P2.p receives some signal through MPK-1 during a period in which VPCs are not receiving inductive signal from the anchor cell.

In a subset of animals, P3.p fails to achieve competence as a VPC, probably because of variable Wnt signal from the posterior during the L2, which contributes to developmental competence of VPCs, while in other animals P3.p is a competent VPC (Euling and Ambros, 1996; Myers and Greenwald, 2007; Eisenmann et al., 1998). Accordingly, in some animals P3.p, P4.p and P5.p exhibit no nuclear MPK-1 (Fig. 6D–F), consistent with P3.p being a competent VPC in these animals and equipotent to its posterior neighbors P4.p and P5.p. In contrast, in other animals, P3.p displays high nuclear MPK-1 levels when its posterior neighbors that are always VPCs, P4.p and P5.p, do not (Fig. 6G–I). We infer that these latter P3.p cells are not competent VPCs.

We observed a similar relationship between non-VPC P9.p and its competent anterior VPC neighbors P7.p and P8.p. Unlike the variability in P3.p competency, P7.p and P8.p are reported to always be competent VPCs in the wild type (Myers and Greenwald, 2007; Kroll et al., 2020). We observed that at the time point where MPK-1 was excluded from the nucleus of P7.p and P8.p, a pool of MPK-1 was observed in the nucleus of P9.p (Fig. S3).

We do not know the nature of signals to non-VPC Pn.p cells at this stage, nor how many of the non-VPC Pn.p cells respond to that signal. However, competent VPCs are presumably refractory in response to this signal, and/or non-VPC Pn.ps have a separate competency program. Additionally, absence vs presence of nuclear MPK-1 in P3.p may be the earliest indicator of competence of P3.p as a VPC.

A phenomenon we observed was decreased expression of tagged HIS-72, an H3 histone, in non-VPC Pn.p cells. The green signal from P2.p was lower than in presumed competent P3.p and P4.p (Fig. 6A), including in P3.p where elevated nuclear translocation of tagged endogenous MPK-1 suggested that the cell was non-competent as a VPC (Fig. 6G). Similarly, the nuclear mNeonGreen::HIS-72 signal of non-VPC P9.p was weaker than that in its lineal homologs P7.p and P8.p (Fig. S3A). We speculate that HIS-72 is expressed at higher levels in VPCs than in non-VPC Pn.p cells to confer differential regulation of gene expression, perhaps related to competence.

### Cytosol-to-nuclear translocation of endogenous MPK-1 is observed in all VPCs

We examined cytosolic-to-nuclear translocation of tagged endogenous MPK-1 in developmental patterning of VPC fates. MPK-1 was originally identified in the VPC system by the ability of reduction-of-function mutations in *mpk-1* to suppress the ectopic multivulva (Muv) phenotype conferred by constitutively activated LET-60 (Wu and Han, 1994; Lackner et al., 1994). Subsequent studies implicated MPK-1 in phosphorylation and repression of the downstream transcription factors LIN-1 (an ETS protein) and LIN-31 (a FoxB protein), which coordinate expression of the homeobox transcription factor LIN-39 and the Mediator complex to induce primary fate (Jacobs et al., 1998; Fantz et al., 2001; Wagmaister et al., 2006a,b; Bagshaw, 1993; Tan et al., 1998; Miller et al., 1996; Underwood et al., 2017).

We failed to observe nuclear translocation of endogenous MPK-1 using the point-scanning Nikon A1 confocal microscope using randomly selected L3 stage animals. We reasoned that a combination of signal too faint to detect and too transient to encounter with regularity among randomly selected L3 animals compromised our efforts at detection. Consequently, we turned to a spinning disk confocal microscope with a more sensitive detector. We also used L3 animals synchronized by the NemaSync filtration device, without the use of hypochlorite treatment and starvation that is typical in the field (see Materials and Methods). With these approaches, we were able to observe exclusion of MPK-1 from VPC nuclei followed by translocation of protein into VPC nuclei. However, detection of nuclear translocation of MPK-1::mKate2 was still at the limit of detection of the instrument, and required deconvolution software to visualize (see Materials and Methods).

**Fig. 7.**
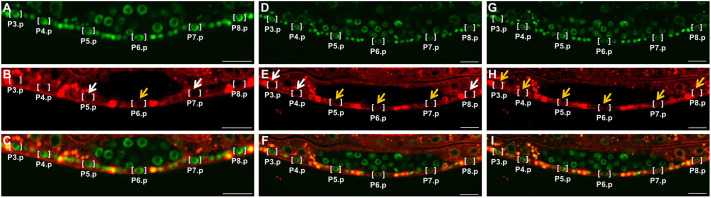
**MPK-1 enters the nuclei of all VPCs, starting with P6.p.** Confocal photomicrographs of tagged endogenous MPK-1 and HIS-72. (A,D,G) Green channel mNeonGreen::HIS-72. (B,E,H) Red channel MPK-1::mKate2. (C,F,I) Merged images. Square brackets indicate Pn.p cells. Orange arrows indicate nuclear MPK-1 signal, white arrows indicate nuclear exclusion of signal, judged primarily by visualization of the nucleolus in the red channel. Left-to-right are three time points. We were unable to reproducibly image one animal continually throughout the time course of VPC induction, because of indicators of desiccation and toxicity. In this series of confocal photomicrographs, the first set of panels (A–C) is a different animal from the second and third sets (D–F and G–I), which are the same animal at different time points. However, these figures are representative of the observed process. The animal in A–C is also enlarged relative to later stages for improved visualization of VPC nuclei. Images are representative of 10 experiments. Scale bars: 10 µm.

Since MPK-1 is necessary and sufficient for induction of primary fate, we reasonably expected to observe nuclear translocation of tagged endogenous MPK-1 only in P6.p, the presumptive primary cell. Unexpectedly, we observed translocation of MPK-1 to the nucleus in all six VPCs (Fig. 7). Our observation indicates that assessment of MPK-1 activation by nuclear translocation is qualitatively different from assessment by the ERK-nKTR reporter described previously (de la Cova et al., 2017).

### Translocation in VPCs occurs earlier and at a higher level in P6.p than in flanking VPCs

The cytosol-to-nuclear translocation of MPK-1::mKate2 occurred in a temporal gradient centered on the source of signal, the anchor cell: first in the Pn.p cell closest to the anchor cell, P6.p (the presumptive primary cell; Fig. 7A–C; Fig. S4), then in P5.p and P7.p (presumptive secondary cells; Fig. 7D–F), and last in P3.p, P4.p and P8.p (presumptive tertiary cells; Fig. 7G–I; Movie 1). This observation indicates that all six VPCs receive the inductive signal from the epidermal growth factor (EGF) protein LIN-3.

**Fig. 8.**
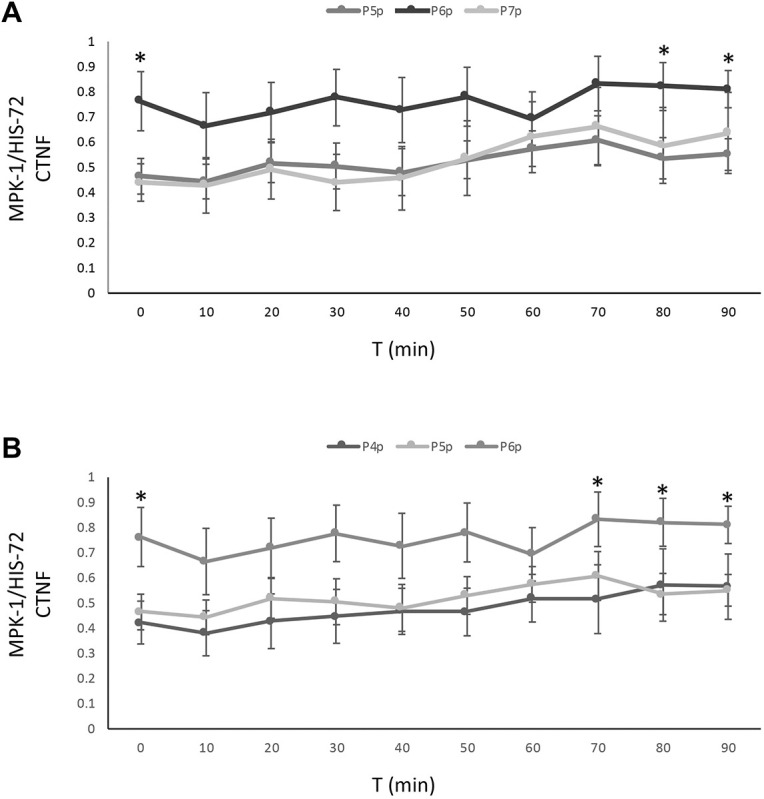
**MPK-1 nuclear localization is higher in P6.p.** (A) A quantification of nuclear MPK-1::mKate2, determined by the ratio of the CTNF between MPK-1::mKate2 and mNeonGreen::HIS-72 is shown for P5.p, P6.p and P7.p. (B) A quantification of nuclear MPK-1::mKate2, determined by the ratio of the CTNF between MPK-1::mKate2 and mNeonGreen::HIS-72 is shown for P4.p, P5.p and P6.p. Data are expressed as mean±s.e.m. (*n*=6). *P<0.05 (one-way ANOVA).

MPK-1::mKate2 is also recruited to P6.p, the presumptive primary cell, at higher levels than surrounding presumptive secondary and tertiary cells. After nuclear translocation was completed in all VPCs, we graphed levels of nuclear MPK-1::mKate2 as a ratio to mNeonGreen::HIS-72. P6.p harbors significantly higher nuclear MPK-1::mKate2 than do the neighboring P5.p and P7.p (*P*=0.02 and 0.05 respectively) (Fig. 8A), or relative to P4.p and P5.p (*P*=0.05; Fig. 8B). Consequently, we conclude that MPK-1::mKate2 translocation into VPCs occurs in both a temporal and a concentration gradient centered on P6.p, the presumptive primary cell. This temporal gradient of nuclear entry of MPK-1::mKate2 into VPCs appears to mirror the hypothetical morphogen gradient of EGF LIN-3 inferred from genetic analyses (Sternberg and Horvitz, 1986; Katz et al., 1995, 1996).

Taken together, our observations of the behavior of endogenous tagged MPK-1 in VPCs suggest unexpectedly rich information encoded in the Ras–Raf–MEK–ERK signal in time and space. This information may reflect the EGF LIN-3 morphogen gradient from the anchor cell, lateral signaling via the Notch protein LIN-12 as part of sequential induction of VPCs, or as-yet unknown feedback loops or parallel signals that regulate the MPK-1 signal at the level of cytosol-to-nuclear translocation.

## DISCUSSION

By tagging the endogenous *C. elegans* ERK protein MPK-1 and tracking its expression and subcellular movements *in vivo*, we have obtained a unique perspective on activation of MPK-1 by upstream signals. We emphasize that this is only one view of ERK activation: antibody detection of the dpMPK-1 phosphorylation or use of ERK-nKTR reporter of substrate phosphorylation are other, complementary, views, though the former may not be feasible in VPCs. However, taken together, these reagents can lead us to a thorough understanding of ERK activation and provide tools to interrogate mechanisms that govern this phenomenon.

As anticipated, we observed that tagged endogenous MPK-1 is expressed ubiquitously. Expression is elevated in the nerve ring, perhaps a reflection of the density of axonal and dendritic projections in ganglia of neurons (Chen et al., 2011). Also, we observed elevated expression in the rectal epithelium, a site of action that has been associated with anti-inflammatory activity in response to a nematode-specific bacterial pathogen, *Microbacterium nematophilum*, but presumably also in preparation for defense against other pathogens (Hodgkin et al., 2013; Anderson et al., 2013). Elevated expression via an extrachromosomal array was previously observed in the rectal epithelium under control of the homeobox transcription factor EGL-5, providing developmental insight into regulation of MPK-1 expression in different tissues (Nicholas and Hodgkin, 2009), and in the present study we observe the same phenomenon with endogenous protein. Expression in the anterior gut, posterior gut and pharynx may also be indicators of an inflammatory mechanism to protect against pathogens.

Endogenous MPK-1 is also expressed throughout the germline. Tagged endogenous MPK-1 allowed us to validate nuclear translocation of MPK-1 as a readout of upstream activation. Occasionally proximal-most oocytes reveal nuclear MPK-1, and this field is expanded in animals mutant for *rskn-1*, a downstream kinase of MPK-1 possibly serving as a negative feedback loop (Arur et al., 2009). In the VPCs, in which dpMPK-1 has not been evaluated because of difficulty in fixation of somatic vs germline structures, we observed translocation of MPK-1 into all six VPC nuclei.

### MPK-1 cytosolic-to-nuclear translocation in the VPCs

The VPCs are a complex system in which at least four signaling cascades are orchestrated to generate the 3°-3°-2°-1°-2°-3° pattern of cell fates with fidelity: core 1°-promoting Ras–Raf–MEK–ERK (Sundaram, 2013) and 2°-promoting Notch (Chen and Greenwald, 2004) signals, coupled with modulatory 1°-promoting PI3K–PDK–Akt (Nakdimon et al., 2012; Shin et al., 2019) and 2°-promoting Ras–RalGEF–Ral–Exo84–GCK-2–MLK-1–PMK-1 signals (Shin et al., 2018; Zand et al., 2011). Additionally, temporal control of VPC patterning is tightly coordinated at the level of the entire animal, probably with the heterochronic system and cell cycle (Wang and Sternberg, 1999; Ambros, 1999; de la Cova et al., 2020). We observed cytosolic-to-nuclear translocation of MPK-1 in all six VPCs in the window during which the VPCs are patterned by LIN-3. We also observed both spatiotemporal and concentration gradients of MPK-1 translocation to the nucleus, centered on P6.p. Our observations are reminiscent of the graded morphogen signal inferred from classic developmental experiments in the vulva (Sternberg and Horvitz, 1986; Katz et al., 1995, 1996). Are the temporal and concentration gradients of MPK-1 translocation, centered on P6.p, a direct reflection of a gradient of growth factor activation of the EGFR receptor protein LET-23? Alternatively, the gradients we observed might reflect the interplay of signals active in naive VPCs, or in parallel to the Ras–Raf–MEK–ERK signal. Our results suggest that regulation of MPK-1 in VPCs is complex, and likely to be subject to a gating phenomenon that restricts the activity to P6.p at precisely the correct time to induce primary fate.

### Regulation of MPK-1 activation

Other signaling axes would be naively expected to extinguish sustained activation of MPK-1 in cells other than P6.p. For example, the lateral signal from the Notch protein LIN-12, in which MPK-1-dependent synthesis of redundant DSL ligands activates LIN-12 in neighboring P5.p and P7.p VPCs to assume secondary fate (Chen and Greenwald, 2004), might be predicted to preclude MPK-1 activation in those cells, or at least activation of substrate transcription factors. A transcriptional client gene of LIN-12, the ERK phosphatase LIP-1, is expressed in P5.p and P7.p as a consequence of lateral signal (Berset et al., 2001). Signaling from LET-23 is similarly thought to be repressed by a receptor tyrosine phosphatase, DEP-1, thus further restricting ERK activation to P6.p as a consequence of initial sequential induction (Berset et al., 2005).

In contrast, we observe that endogenous MPK-1 enters the nuclei of all six VPCs, which implies that all six receive inductive signal via the LET-60 (Ras)–LIN-45 (Raf)–MEK-2 (MEK)–MAPK cascade. In contrast to our results with tagged endogenous MPK-1, the ERK-nKTR reporter of ERK activation of substrates suggested that MPK-1 was activated only in P6.p during the L3 stage. (The ERK-nKTR marker was also active in pulsatile waves prior to induction, in the L2 stage, hinting that some form of ‘pre-patterning’ occurs prior to induction; de la Cova et al., 2017). Although ectopically expressed, this reporter is single copy and hence unlikely to be subject to undesirable effects of overexpression, and is well validated elsewhere in the animal. Furthermore, activation of the ERK-nKTR biomarker closely resembles what we would expect from such a reporter predicted by the genetics: activation restricted to P6.p during the window in which P6.p is induced by the Ras–Raf–MEK–ERK cascade.

In addition to contradiction by the results using the ERK-nKTR reporter, our observation conflicts with transcriptional reporter analysis of genes reported to be responsive to MPK-1 primary-promoting signaling. During VPC induction, the promoter of *egl-17* drives expression of CFP in an abbreviated gradient: expression is strong and sustained in P6.p but weak and transient in P5.p and P7.p. The transient expression in P5.p and P7.p is a result of LIN-12-dependent expression of the LIP-1 phosphatase and other lateral signaling target genes (Yoo et al., 2004). Another reporter, the promoter of *lag-2* driving expression of YFP, is expressed only in primary lineages (Zhang and Greenwald, 2011).

We surmise that the discrepancy between the two methods of measurement reveals overlapping systems of regulation of MPK-1 to keep its activation constrained both spatially and temporally. At least at the level of translocation of MPK-1::mKate2, activation of MPK-1 appears to be independent of LIN-12 or expression of LIP-1. So, there must be at least one other mechanism for gating the activity of MPK-1. Could output of MPK-1 be regulated through a series of interactions with transcription factors? This interpretation is unlikely, given that ERK-nKTR is a reporter of direct phosphorylation of substrate, and thus reflects activation of MPK-1 upstream of transcription factors (de la Cova et al., 2017; Regot et al., 2014). But possibly the ERK-nKTR reporter does not represent dpMPK-1, just the ability to phosphorylate a specific, defined substrate. Or perhaps MPK-1 is rapidly dephosphorylated in all but P6.p, although it is not accompanied by nuclear export, but signaling to downstream LIN-1 and LIN31 transcription factors is nonetheless incapacitated.

### General MAPKs

Other subfamilies of MAPKs may share regulatory mechanisms. Our laboratory documented that tagged endogenous p38 MAPK PMK-1, the known endpoint of the Ral secondary-promoting modulatory signal, is partially nuclear in every somatic cell of the animal (Shin et al., 2018; expression was probably silenced in the germline). This result was not observed with transgenic overexpression of GFP-tagged PMK-1 (Mertenskötter et al., 2013; our unpublished results), perhaps because of unfavorable signal-to-noise ratio attendant upon overexpression. Like ERK, p38 MAPKs are also expected to undergo nuclear translocation upon activation (Ben-Levy et al., 1998). But perhaps a live animal experiences tonic, low-level activation of inflammatory responses mediated by PMK-1, maybe as a preventative measure against an environment with many pathogens. Thus, a small pool is always recruited to nuclei, but this pool may be miniscule compared with overexpressed heterologous protein, and so is lost due to infelicitous signal-to-noise ratio.

Still other subfamilies of MAPKs use nuclear translocation as a step in their activation of nuclear targets. JNK MAPKs translocate upon activation (Liu et al., 1996). The fourth subfamily of MAPKs, the non-canonical ERK5 with its transactivation domain that regulates transcription, is also regulated by translocation to the nucleus upon activation by upstream cascades (Gomez et al., 2016). Thus, this regulatory modality of MAPKs is well established.

Importantly, we note that many MPK-1 targets are not transcription factors or other proteins occupying the nucleus, and thus are not subject to nuclear translocation of MPK-1 as a biomarker for activation. This is clearly true for MPK-1 substrates in the germline, where myriad non-nuclear substrates have been identified and dpMPK-1 detected via antibody staining of fixed, extruded gonads (Arur et al., 2009). But non-nuclear substrates throughout the animal are also likely to be subject to phosphorylation by MPK-1. This phenomenon restricts the utility of tagged endogenous MPK-1 to events in the nucleus.

Our analysis points to nuclear translocation of endogenous MPK-1 as a robust system for analyzing activation in a live animal. This includes transient activation during developmental patterning of VPC fate, which was detected only at the lower limit of sensitivity of our instrument. We also conclude that the VPCs are a system where multiple levels of regulation of ERK are employed to achieve the desired developmental outcome. Although outside the scope of this analysis, nuclear translocation of endogenous MPK-1, the ERK-nKTR reporter, and perhaps antibody detection of dpMPK-1 could be deployed in concert to disentangle relationships between different modalities of regulation of ERK activation in an active developmental system. In the end there can be only one: P6.p assumes primary fate in 99.8% of wild-type animals (Braendle and Félix, 2008; Shin et al., 2019). Layers of regulation of ERK, including control of nuclear translocation, may impose strictures that contribute to this level of developmental fidelity.

## MATERIALS AND METHODS

### *C. elegans* handling and genetics

All strains were derived from the wild-type Bristol N2 parent strain and grown under standard conditions at 20°C unless stated otherwise (Brenner, 1974). Nomenclature conforms to that of the field (Horvitz et al., 1979). All crosses were performed using standard methods, available upon request. Genotypes of strains used in this study are listed in Table S3.

### Plasmids and generation of CRISPR strains

Details of plasmid constructions are available upon request. Primers used in this study are listed in Table S4, and plasmids in Table S5. The *mpk-1(re171[mpk-1::mkate2^SEC^3xFlag])* and *mpk-1(re172[::mKate2^3xFlag])* alleles were generated using the self-excising cassette (SEC) approach of positive and negative selection for CRISPR inserts (Dickinson et al., 2015). Small guide RNAs (sgRNAs) are listed in Table S6. The repair template for *mpk-1* was generated with primers oNR059, oNR060 and gBlock oNR067 for cloning into plasmid pDD285. We microinjected a mix of pCFJ104 (10 ng/µl), pNR9 (50 ng/µl), pNR10 (50 ng/µl), pNR11 (10 ng/µl) into N2 animals. Edited animals were identified by resistance to hygromycin (HygR; 5 mg/ml in filtered ddH_2_0, added directly to plates) and the Rol phenotype of the *sqt-1(*d*)* marker, both contained in the SEC. Homozygous animals were viable as this was C-terminal insertion. Selected animals were subsequently heat-shocked to induce expression of Cre, also contained in the SEC. Successful removal of the SEC was indicated by loss of the Rol marker (Dickinson et al., 2015). Triplex PCR detection primers oNR094, oNR095 and oNR096 were used to confirm insertion and to sequence regions of homology subject to homology-directed repair (HDR). Single and pooled animal genotyping PCR reactions used Taq PCR Master Mix (Qiagen).

### Fluorescence imaging and quantification of relative nuclear MPK-1 levels in VPCs

For all imaging, animals were mounted live in M9 buffer containing 2% tetramisole on slides with a 3% agar pad. Differential interference contrast (DIC)/Nomarski optics and fluorescence microscopy were captured using a Nikon A1si confocal microscope with 488 nm and 561 nm lasers (Figs 2–5; Figs S1 and S2) or a Yokogawa CSU-W1 spinning disk confocal laser microscope with 488 nm and 561 nm lasers and Photometrics Prime BSI camera (Figs 6 and 7; Figs S3 and S4). Slides prepared for spinning disk time-lapse imaging were sealed with Vaseline, lanolin and paraffin (VALAP) to prevent animals from drying out. Captured images were processed using NIS Elements Advanced Research, version 4.40 software (Nikon). Additional deconvolution processing was performed on all time-lapse images within the Nikon Elements software utilizing the 3-D Richardson–Lucy algorithm over 35 iterations.

To determine relative levels of nuclear localization of MPK-1, animals were imaged in 10-min intervals 24 h post synchronization. Following deconvolution (above), fluorescent intensity measurements for both MPK-1::mKate2 and mNeonGreen::HIS-72 were recorded for each P4.p–P8.p nucleus using NIS Elements Advanced Research, version 4.40. Corrected total nuclear fluorescence (CTNF) was calculated by subtracting background fluorescence. To account for variations, we then divided the MPK-1::mKate2 CTNF intensity by the corresponding mNeonGreen::HIS-72 intensity. *P*-values were calculated using one-way ANOVA.

### Immunoblotting

For preparation of protein lysates, animals were washed from plates and boiled in 4% SDS loading buffer at 95°C for 2 min. Lysates were separated on a 4–15% SDS gel (BIO-RAD), transferred to an Immobilon PVDF membrane (EMD Millipore) and probed with the following antibodies: monoclonal mouse anti-Flag antibody (Sigma-Aldrich, F1804) and monoclonal mouse anti-α-tubulin antibody (Sigma-Aldrich, T6199) diluted 1:2000 in blocking solution overnight. Following the primary incubation, blots were incubated with goat anti-mouse-IgG horseradish peroxidase-conjugated secondary antibody (MilliporeSigma, 12-349), diluted 1:5000 in blocking solution for 1 h. Immunoblots were then developed using an ECL kit (Thermo Fisher Scientific) and X-ray film (Phenix).

### Synchronized populations

To achieve tightly synchronized populations without potential biological artifacts introduced by a bleach or starvation synchronization protocol, we utilized the NemaSync *C. elegans* synchronizer model 5000 (InVivo Biosystems). Mixed-stage animals were grown to high density on 12–20 10 mm nematode growth medium plates seeded with *Escherichia coli* OP50. Animals were then washed off in M9 buffer and added to the stabilization filter, allowing gravid adults to be separated from all other stages. Adult animals were collected from the filter and pipetted onto the harvest filter. Recently hatched L1 larvae collected from 15-min windows were then re-plated onto OP50-seeded plates and grown at 20°C for precisely the desired time. All reported times for time-lapse imaging refer to time post-plating of synchronized L1 animals.

## Supplementary Material

Supplementary information

Reviewer comments
