# Supplementary material for: Nuclear translocation of the tagged endogenous MAPK MPK-1 denotes a subset of activation events in C. elegans development
Source: J Cell Sci. 2021 Sep 3;134(17):jcs258456. doi: 10.1242/jcs.258456 (PMC8445601; doi:10.1242/jcs.258456)
Supplement: Supplementary information [file joces-134-258456-s1.pdf]

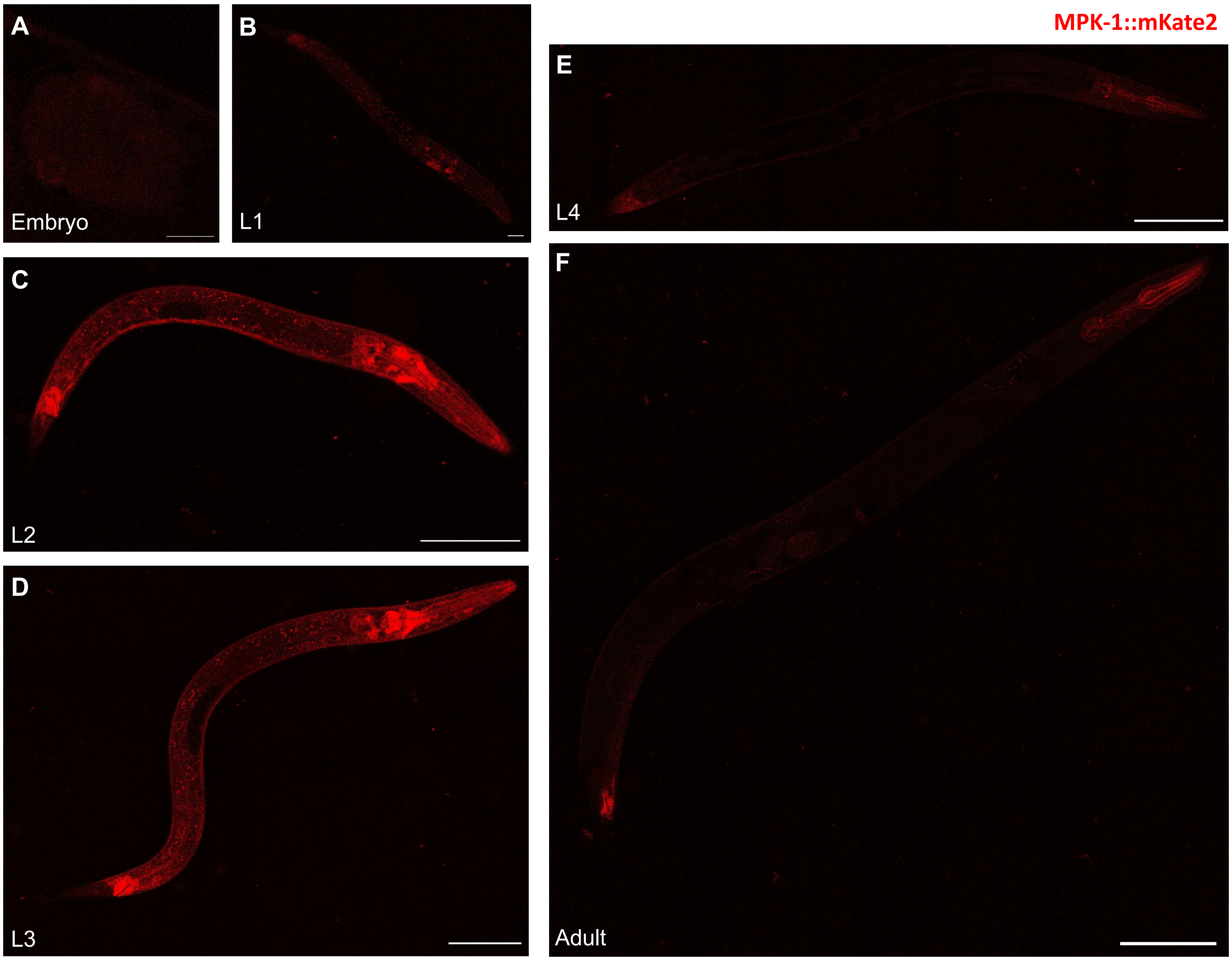

Fig. S1. Endogenous MPK-1 is expressed throughout the animal and is elevated in certain tissues and at certain stages. Confocal micrographs of MPK-1::mKate2 animals at different stages of the life cycle. ****All images were captured with the same exposure levels.***** **A)** An early stage embryo in the uterus of its mother with low background MPK-1. **B)** L1 larva. **C)** L2 larva evincing global elevation of MPK-1 expression and increased expression in the pharynx, anterior intestine and posterior intestine. **D)** L3 larva with similarly elevated expression. **E)** L4 larva with decreased global expression but relative expression still elevated in certain tissues. **F)** Adult animal with decreased expression except in rectal epithelia and the pharynx. Scale bars = 10 µm (**A,B**), 50 µm (**C,D,E**), and 100 µm (**F**).

Journal of Cell Science • Supplementary information

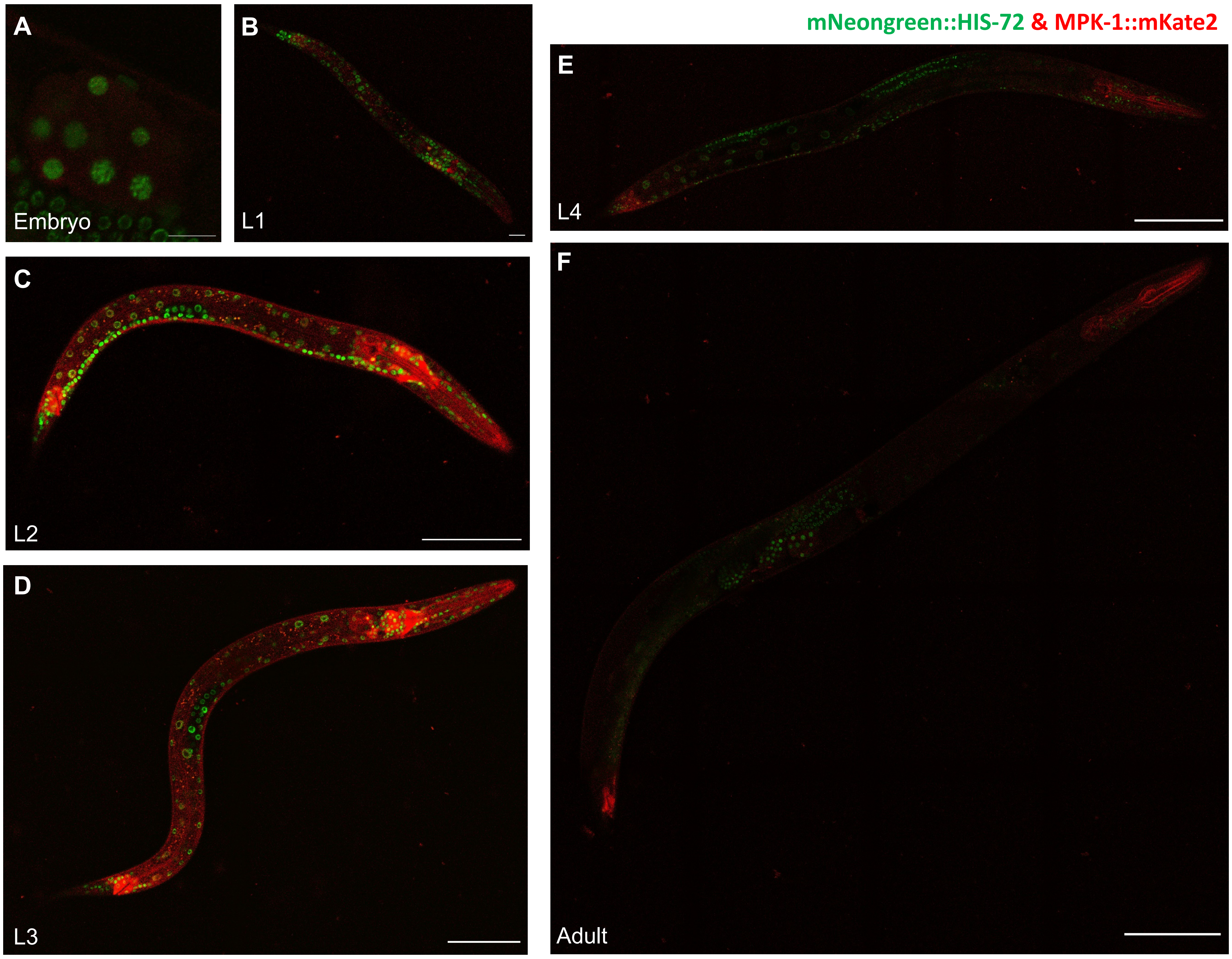

Fig. S2. Endogenous MPK-1 with a nuclear marker as a reference point. The same images as in Supplementary Figure 1 but with mNeonGreen::HIS-72 nuclear marker in green. **A)** An early stage embryo in the uterus of its mother. **B)** L1. **C)** L2. D) L3. **E)** L4. **F)** Adult animal. Scale bars = 10 µm (A,B), 50 µm (**C,D,E**), and 100 µm (**F**).

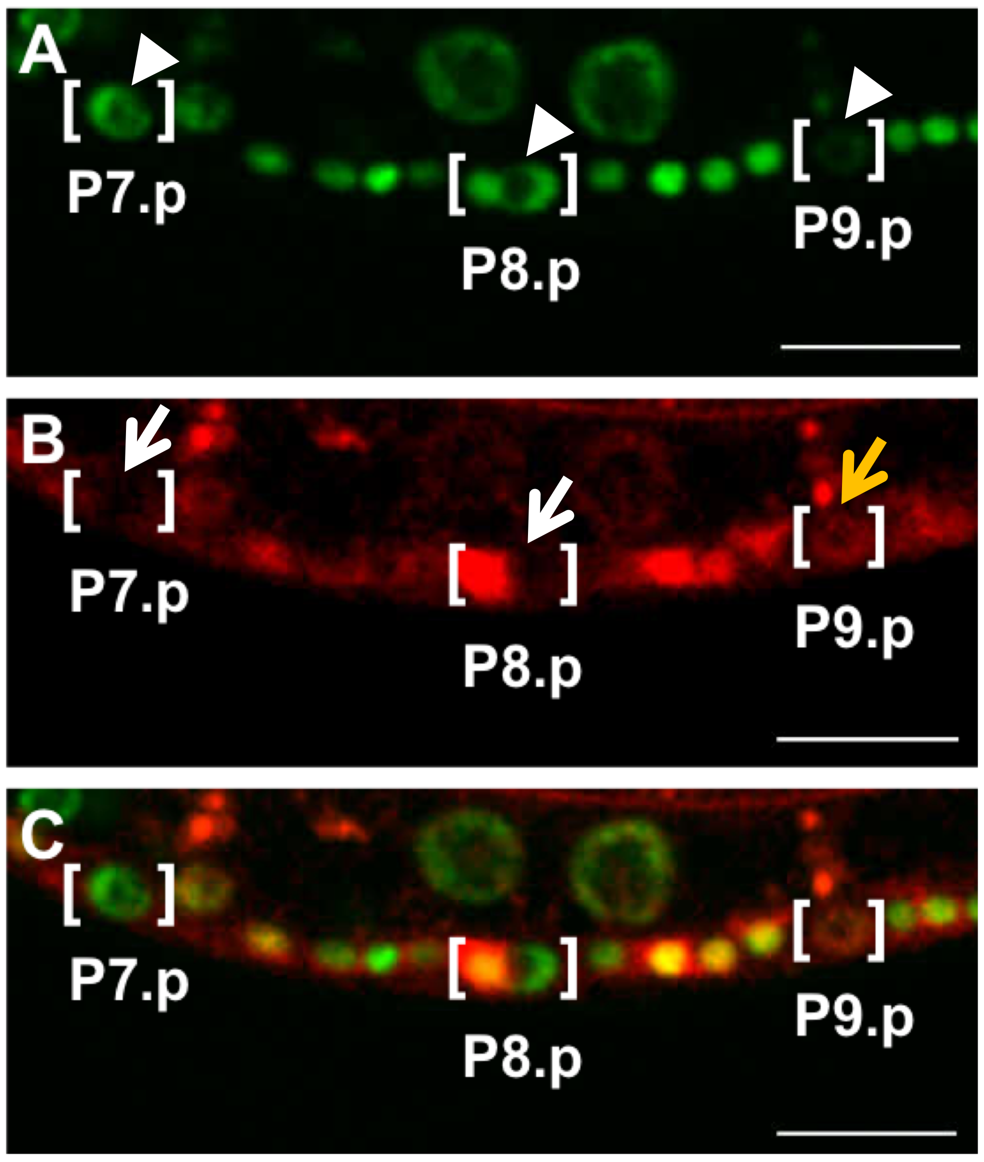

Fig. S3. Tagged MPK-1::mKate2 translocates to the nucleus of posterior non-VPC P9.p prior to induction of VPCs P7.p and P8.p. **A)** Green channel nuclei, mNeonGreen::HIS-72. **B)** Red channel MPK-1::mKate2. A ventral cord neuron (bright red puncta) overlaps with the P8.p nuclei **C)** Merged images. Brackets are included around nuclei for references. Many but not all such nuclei have nuclear MPK-1 (smaller ventral nuclei in the figure). Orange arrows indicate nuclei with nuclear MPK-1, white arrows nuclei without nuclear MPK-1. The white arrowhead indicates VPC and non-VPC nuclei with differing levels of mNeonGreen::HIS-72 intensity. Scale bars = 10 µm.

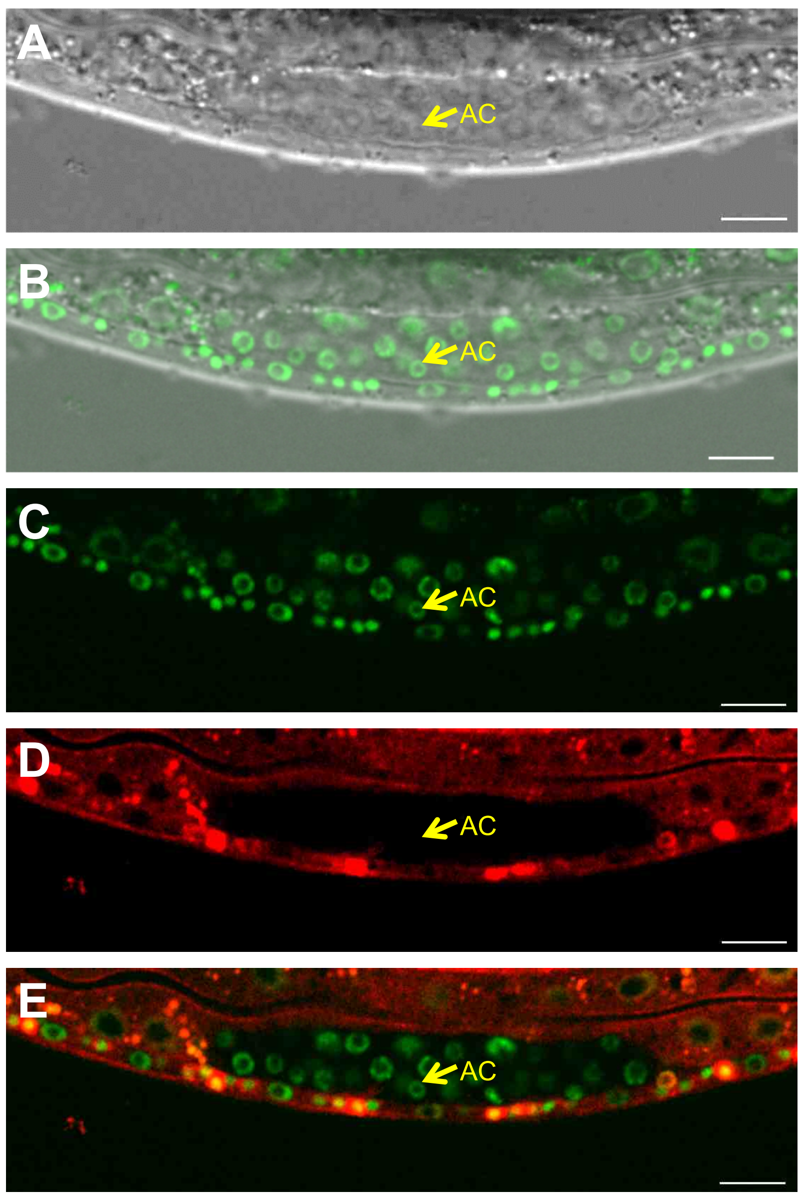

Fig. S4. The gradient of relocalization of MPK-1::mKate2 to the nucleus centers on P6.p, the VPC closest the the Anchor Cell in the developing ventral somatic gonad. All spinning disk confocal photomicrographs are of the same animal. **A)** Location of the AC by DIC and **B,C)** merge and mNeonGreen::HIS-72, respectively. **D)** MPK-1::mKate2 and **E)** merge. Yellow arrows point to the AC. Scale bars = 10 µm.

Journal of Cell Science • Supplementary information

Table S1.

Genotype:	WT	*mpk-1(re172)*
L1/L2 Healthy Animals	380	365
Unhatched	0	0
L1/L2 rods	0	0

Table S2.

Genotype:	WT	*mpk-1(re172)*
Normal Vulvae	59	52
Vulvaless	0	0
Ectopic Pseudovulvae	0	0

Table S3. Strains

Strain	Genotype
DV3261	*mpk-1(re171[mpk-1::mKate2^SEC^3xFlag])* III
DV3262	*mpk-1(re172[mpk-1::mKate2^3xFlag])* III
DV3285	*his-72(cp76[mNeonGreen^3xFlag::his-72]), mpk-1(re172[mpk-1::mKate2^3xFlag])* III
BS3760	*rskn-1(ok159)* I
DV3317	*rskn-1(ok159)* I; *his-72(cp76[mNeonGreen^3xFlag::his-72]), mpk-1(re172[mpk-1::mKate2^3xFlag]*) III

Table S4. Primers

Name	Sequence	Used for
oNR065	GGCTCATCACAGACGAATGGGTTTAAGAGCT ATGCTGGAAACAG	mpk-1 sgRNA-1 mutagenesis
oNR066	AGCTTTTCAGCGGGAACGGGGTTTAAGAGCT ATGCTGGAAACAG	mpk-1 sgRNA-2 mutagenesis
oNR023	CAAGACATCTCGCAATAGG	mpk-1 sgRNA mutagenesis
oNR059	CGTGATTACAAGGATGACGATGACAAGAGA ATTAGTAGTATTTACCCACTAAATTAG	mpk-1 homology arm-2 FW
oNR060	GGAAACAGCTATGACCATGTTATCGATTTCC GCTATGATTTGTTGTGTAAACC	mpk-1 homology arm-2 RV
oNR067	CGATTTTCAGTATCGACATCGAGCAAGCATT GGCTCACCCATACTTGGAGCAATACTACGAT CCAGGAGATGAGCCAGTTTGTGAGGAACCAT TCACTTTGGAAATGGAATTCGACGATTTACC GAAGGAGAAGCTGAAGGAGCTGATTTGGGA AGAAGCCGAGGCTCATCACAGACGAATGGA GGCAGAAGCGGCTGCAAGGAATAATGGAGG GCAGAATCCTGTTTCAGCCGGAGGTAGCGCC GGCGGAAGTGCTGGTGGAATGGTCTCCGAGC TCATTAAAGAAAACATG	mpk-1 homology arm-1 gBlock
oNR094	ACCAAAACAACCATGGGCTCG	mpk-1 genotyping FW
oNR095	GCTCCAAGTATGGGTGAGCC	mpk-1 genotyping RV-1
oNR096	GGTTCCCTCGTATGGCTTTCC	mpk-1 genotyping RV-2

Journal of Cell Science • Supplementary information

Table S5. Plasmids

Name	Description	Used for
pNR9	Cas9 and MPK-1 sgRNA-1	MPK-1 sgRNA-1
pNR10	Cas9 and MPK-1 sgRNA-2	MPK-1 sgRNA-2
pNR11	MPK-1::mKate2^3xFlag	SEC CRISPR plasmid repair template
pCFJ104	$P_{myo\text{-}3}$::*mCherry*	Co-injection marker
pJW1236	Cas9 and sgRNA expression plasmid	mpk-1 SEC-CRISPR tagging
pDD285	mKate2::3xFlag SEC repair template	mpk-1 SEC CRISPR repair template

Table S6. sgRNA sequences and PAMs

Sequence	Used for
GGCTCATCACAGACGAATGG	mpk-1 SEC-CRISPR tagging
AGCTTTTCAGCGGGAACGGG	mpk-1 SEC-CRISPR tagging

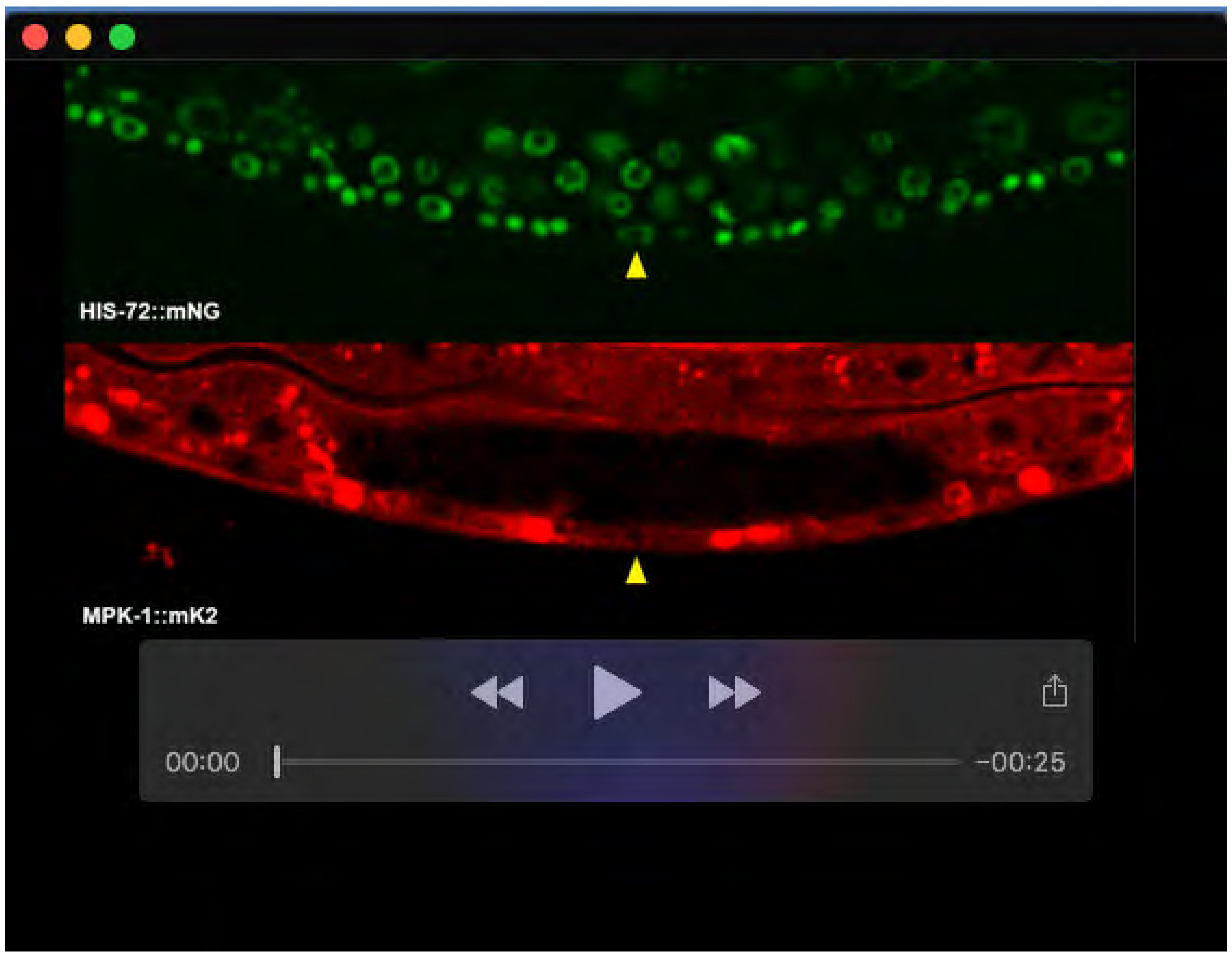

Movie 1. Time lapse film of MPK-1::mKate2 entering P6.p first. Arrows indicate P6.p and other VPCs.

Journal of Cell Science • Supplementary information
