# Supplementary material for: Nuclear translocation of the tagged endogenous MAPK MPK-1 denotes a subset of activation events in C. elegans development
Source: J Cell Sci. 2021 Sep 3;134(17):jcs258456. doi: 10.1242/jcs.258456 (PMC8445601; doi:10.1242/jcs.258456)
Supplement: Reviewer comments [file joces-134-258456_review_history.pdf]

Nuclear translocation of the tagged endogenous MAPK MPK-1 denotes a subset of activation events in *C. elegans* development

Neal R. Rasmussen and David J Reiner
DOI: 10.1242/jcs.258456

Editor: Guangshuo Ou

Review timeline
Original submission: 22 January 2021
Editorial decision: 15 March 2021
First revision received: 4 May 2021
Accepted: 14 July 2021

Original submission

First decision letter

MS ID#: JOCES/2021/258456

MS TITLE: Nuclear translocation of tagged endogenous ERK/MPK-1 MAP Kinase denotes a subset of activation events in C. elegans development

AUTHORS: Neal R Rasmussen and David J Reiner
ARTICLE TYPE: Research Article

We have now reached a decision on the above manuscript.

To see the reviewers' reports and a copy of this decision letter, please go to: https://submit-jcs.biologists.org and click on the 'Manuscripts with Decisions' queue in the Author Area. (Corresponding author only has access to reviews.)

As you will see, the reviewers gave favourable reports but raised some critical points that will require amendments to your manuscript. I hope that you will be able to carry these out by textual changes and discussions to Reviewer #1's concerns, because I would like to be able to accept your paper.

We are aware that you may be experiencing disruption to the normal running of your lab that makes experimental revisions challenging. If it would be helpful, we encourage you to contact us to discuss your revision in greater detail. Please send us a point-by-point response indicating where you are able to address concerns raised (either experimentally or by changes to the text) and where you will not be able to do so within the normal timeframe of a revision. We will then provide further guidance. Please also note that we are happy to extend revision timeframes as necessary.

Please ensure that you clearly highlight all changes made in the revised manuscript. Please avoid using 'Tracked changes' in Word files as these are lost in PDF conversion.

I should be grateful if you would also provide a point-by-point response detailing how you have dealt with the points raised by the reviewers in the 'Response to Reviewers' box. Please attend to all of the reviewers' comments. If you do not agree with any of their criticisms or suggestions please explain clearly why this is so.

© 2021. Published by The Company of Biologists under the terms of the Creative Commons Attribution License (https://creativecommons.org/licenses/by/4.0/).

Reviewer 1

Advance summary and potential significance to field

This manuscript reported the nucleus localization of endogenous ERK/MPK-1 MAP Kinase during C. elegans development. The authors used mKate2-3×Flag as a fluorescent and biochemical tag to label endogenous MPK-1 by CRISPR-Cas9 based genome editing and verified that the fusion protein works well by biochemistry and imaging, demonstrating its functionality. They further examined the localization of mKate2-3×Flag tagged endogenous MPK-1 during maturing oocytes and validate its nuclear translocation as a reporter of certain activation events. They also showed that mKate2-3×Flag tagged endogenous MPK-1 is localized in the nucleus of all the six VPCs. This finding is interesting and appears to be different from the previous results using other ERK-nKTR reporter systems. The study provides a useful tool for the community.

Comments for the author

While the localization of MPK-1 is interesting, the current work does not provide a necessary advance for publication in the JCS, which requires mechanistic insights to a certain level. In other words, a simple description of a kinase localization, though valuable, is probably insufficient.

Other major issues:

1. It remains unclear whether mKate-tag works well for tagging each protein functionally. Because of the apparent discrepancy from the previous results, the major conclusion of MPK-1 localization should be backed up by alternative approaches, including immunofluorescence, or GFP-tagging.

2. They should study mKate2-3×Flag tagged endogenous MPK-1 in VPCs localization in the mutants of Ras>Raf>MEK>ERK cascade and LIN-12/Notch signaling pathway which may provide some mechanistic insights into the function or nuclear location of MPK-1.

Minor corrections:

In figure 3 and figure 4 of HIS-72::mNeonGreen::3xFlag, the figure legend is mNG::HIS-72

The figure 5 legend, 'Scale bars = 10 µM' should be 'Scale bars = 10 µm'.

Reviewer 2

Advance summary and potential significance to field

This paper reports what promises to be a highly useful reagent for studying endogenous MAPK signaling in C. elegans. The marker, a knocked in tagged MAPK protein, is validated in not only oogenesis but also the well -studied vulval development. Progress in the analysis of this paradigmatic case of developmental signaling and signal transduction has lagged in recent years due to lack of tools; this new reagent may well juice up the field!

Comments for the author

The methods are clearly explained, and as far as I can tell, all the reagents are clearly defined. The logic is clear and figures are well presented. The results with this reagent differ somewhat, as clearly explained in the text, from previous reagents. The questions raised by the discrepancy seem interesting, and I have no reason to doubt these current results. While there are many possible experiments that could probe the system further, I think they are beyone the scope of this already information dense, paper. I thus recommend publication as is.

© 2021. Published by The Company of Biologists under the terms of the Creative Commons Attribution License (https://creativecommons.org/licenses/by/4.0/).

First revision

Author response to reviewers' comments

Point-by-point response to reviewer comments. Text and table additions in the manuscript are marked in green. Responses to reviewers is written in blue, below. We also took the opportunity to clean up consistency of nomenclature of CRISPR-based tags in the manuscript.

Reviewer #1:

While the localization of MPK-1 is interesting, the current work does not provide a necessary advance for publication in the JCS, which requires mechanistic insights to a certain level. In other words, a simple description of a kinase localization, though valuable, is probably insufficient.

We ask that the reviewer consider that we do present a mechanistic insight of a technological nature: the nKTR reporter of phosphorylation substrate can yield a markedly different answer in live animals than does nuclear translocation of endogenous protein. Not only does this observation, like most good science, raise more questions than it answers, it holds methodological importance for the target audience interested in questions of cell signaling.

It remains unclear whether mKate-tag works well for tagging each protein functionally. Because of the apparent discrepancy from the previous results, the major conclusion of MPK-1 localization should be backed up by alternative approaches, including immunofluorescence, or GFP-tagging.

This is an excellent point raised by the reviewer! We have quantified, using animals harboring tagged MPK-1, completion of two developmental events governed by MPK-1: induction of the excretory duct cell, whose absence confers rod-like larval lethality at the L1 and L2 stages, and Vulvaless (Vul) or Multivulva (Muv) phenotypes, caused by under- or over-induction during the VPC patterning process. In this we followed the lead of a fine recent study by Gauthier and Rocheleau, *Development* (2021). Using these assays, the authors found that the *re202* tag of endogenous LET-23/EGFR, provided to them by us, caused low-penetrance Vul and Muv phenotypes, consistent with mild reduction of function. The same study found that tags of LIN-2, LIN-7, and LIN-10 did not detectably perturb function. Our own study (Shin *et al. Cell Reports*, 2018) similarly evaluated functions of tagged RAL-1/Ral, GCK-2/MAP4K and PMK-1/p38 MAP kinase, finding that none detectably changed function. These data for MPK-1::mKate2 were added as Supplementary Tables 5&6, and are supported by a new paragraph with callouts in the first section of the Results. Thus, we conclude that the tagged MPK-1 used in this study is functional.

They should study mKate2-3×Flag tagged endogenous MPK-1 in VPCs localization in the mutants of Ras>Raf>MEK>ERK cascade and LIN-12/Notch signaling pathway, which may provide some mechanistic insights into the function or nuclear location of MPK-1.

We very much agree that the next step is to compare and contrast MPK-1::mKate2 and ERK- nKTR reporters in developing animals in response to genetic manipulations of the signaling system in VPC patterning. Unfortunately, we think that such an undertaking, with extensive spinning disk filming and image analysis, would be extremely demanding and comprise an independent paper. Reviewer #2 appears to agree with this assessment: “While there are many possible experiments that could probe the system further, I think they are beyone <sic> the scope of this, already information dense, paper. I thus recommend publication as is.”

Second decision letter

MS ID#: JOCES/2021/258456

MS TITLE: Nuclear translocation of tagged endogenous MPK-1/ERK denotes a subset of activation events in C. elegans development

© 2021. Published by The Company of Biologists under the terms of the Creative Commons Attribution License (https://creativecommons.org/licenses/by/4.0/).

AUTHORS: Neal R Rasmussen and David J Reiner

ARTICLE TYPE: Research Article

I am happy to tell you that your manuscript has been accepted for publication in Journal of Cell Science, pending standard ethics checks.

© 2021. Published by The Company of Biologists under the terms of the Creative Commons Attribution License (https://creativecommons.org/licenses/by/4.0/).
